# Objective Analysis of Reading Ability Using an Eye Tracker in Intermittent Exotropia

**DOI:** 10.3390/life15111778

**Published:** 2025-11-20

**Authors:** Dong Hyun Kim, Jeong-Min Hwang, Hee Kyung Yang

**Affiliations:** 1 Department of Ophthalmology, Seoul National University College of Medicine, Seoul National University Bundang Hospital, Seongnam 13620, Republic of Korea; himrdh@gmail.com; 2 Department of Ophthalmology, Strabismus & Pediatric Ophthalmology Center, Kim’s Eye Hospital, 136, Yeongsin-ro, Yeongdeungpo-gu, Seoul 07301, Republic of Korea

**Keywords:** reading ability, eye tracker, intermittent exotropia

## Abstract

**Background/Objectives**: This study’s objective was to analyze the reading ability in patients with intermittent exotropia using an eye tracker and determine how the clinical characteristics of intermittent exotropia may affect reading ability. **Methods**: We compared the reading speed (LPS; letters per second) of 25 intermittent exotropia patients to 25 age-matched normal controls who were 13 years old or older with best-corrected visual acuities of ≥20/25. Correlations between reading ability and clinical characteristics of intermittent exotropia were evaluated. **Results**: Reading speed was significantly slower in the intermittent exotropia group (6.1 ± 0.81 LPS) than in the control group (6.8 ± 1.11 LPS, *p* = 0.014). The Newcastle control scores, Mayo Clinic office-based scale and the patients’ deviation angle were not significantly related to reading speed (*p* = 0.132, 0.197, and 0.807, respectively). Fixation disparity score measured with an eye tracker during the reading task showed a statistically significant negative correlation with reading speed (Spearman’s rank correlation coefficient = −0.458, *p* = 0.028). **Conclusions**: Reading speed was slower in patients with intermittent exotropia compared to age-matched controls without strabismus. Only objective fusional control scores measured with an eye tracker showed significant correlation with the reading speed.

## 1. Introduction

Intermittent exotropia is the most common type of strabismus in East Asian populations, including South Korea, China, and Japan, and its prevalence has been consistently reported in epidemiologic studies of children and adults [1,2,3,4,5]. Although intermittent exotropia is frequently encountered in clinical practice, patients and their caregivers often express concerns about how intermittent exotropia may influence visual function and whether it could interfere with learning and academic development. Despite this concern, the existing body of evidence is limited, and clinicians often struggle to provide comprehensive, evidence-based answers.

Reading ability is crucial for acquiring new knowledge and achieving academic progress [6]. Reading skills in early life could be a good indicator of future academic performance [7,8]. Recent investigations have begun to explore this relationship in intermittent exotropia. Fang et al. [9] reported that school-aged children with intermittent exotropia exhibited slower reading speeds compared to peers with normal alignment, while Yang et al. [10] showed that visual-motor integration and attentional deficits further contributed to impaired reading ability. Hirota et al. [11,12] highlighted that poor binocular coordination during smartphone reading correlated with increased monocular viewing and slower performance. Together, these findings suggest that intermittent exotropia may exert multifaceted effects on reading and related cognitive functions.

However, most available studies are limited to pediatric cohorts and often rely on subjective measures of fluency or comprehension rather than objective assessments of binocular coordination. Subjective reports, while valuable, are influenced by recall bias, differences in parental perception, and variability in children’s willingness to cooperate during clinical testing. If we had more concrete data on the effect of intermittent exotropia on reading, we could give better evidence-based answers. Eye trackers, which enable real-time measurement of fixation disparity and gaze dynamics, provide a more precise tool for understanding the mechanisms underlying reading impairment in intermittent exotropia.

The purpose of our study was to objectively analyze the reading ability in patients with intermittent exotropia compared with age-matched controls, using an eye tracker as the primary measurement tool. We aimed not only to quantify differences in reading speed but also to examine how clinical characteristics—such as fusional control, deviation angle, and stereopsis—correlate with functional outcomes.

## 2. Materials and Methods

### 2.1. Participants

A total of 50 individuals were recruited for this prospective study. Recruitment occurred through direct invitation from the outpatient strabismus clinic and voluntary participation. Participants were required to be at least 13 years of age to ensure they had attained an appropriate level of reading fluency for standardized testing. To exclude confounding visual deficits, best-corrected visual acuity (BCVA) of 20/25 or better in both eyes was mandatory. Participants were divided into two groups: the intermittent exotropia group, consisting of patients demonstrating an exodeviation greater than 10 prism diopters (PD) at near fixation; and the control group, composed of orthotropic individuals or those with minor exophoria of less than 5 PD.

Patients with amblyopia manifesting as an interocular difference in BCVA of two lines or greater were excluded. Individuals with paralytic or restrictive exotropia, sensory exotropia, vertical deviations greater than 5PD, or dissociated vertical deviation were also excluded. Ocular pathologies, such as retinal disease, optic neuropathy, or keratopathy, were also excluded, as were systemic or neurological disorders, including myasthenia gravis, chronic progressive external ophthalmoplegia, and intracranial lesions that could affect ocular motility. Detailed ophthalmic histories were obtained to screen for additional exclusion factors such as previous ocular surgery, congenital anomalies, or systemic illnesses affecting eye movements.

All participants underwent a complete ophthalmic examination prior to enrollment to verify eligibility. The study protocol was reviewed and approved by the Institutional Review Board of Seoul National University Bundang Hospital (B-1909-562-303), and all participants provided written informed consent. The research was conducted in accordance with the principles of the Declaration of Helsinki.

### 2.2. Ophthalmic Examination

Each subject received a comprehensive eye examination from an experienced pediatric ophthalmologist. The angle of deviation was measured using the prism and alternate cover test at near (0.3 m) and distant (6 m) fixation. Measurements were taken while the participants wore their optimal refractive correction. Cycloplegic refraction was determined after instilling 1% cyclopentolate hydrochloride, and spherical equivalents were calculated for analysis.

The near point of accommodation (NPA) was assessed monocularly using the push-up method. Participants focused on an “E” target slightly above their near-threshold acuity. The target was gradually advanced until the participants reported sustained blur. Each eye was tested three times, and the mean value was used for statistical comparisons. The near point of convergence (NPC) was evaluated binocularly using an accommodative rule. A small fixation target was slowly advanced until the participant reported diplopia or the examiner observed one eye diverging. Three measurements were averaged again [13].

Stereopsis was assessed using the Randot stereotest (Stereo Optical Company, Chicago, IL, USA) and converted into logArcsec equivalents.

### 2.3. Reading Task and Eye-Tracking Protocol

Reading performance was assessed using an infrared eye-tracking system (Tobii Technology, Stockholm, Sweden) integrated with a 16-inch monitor. The monitor had a resolution of 1920 × 1080 pixels and was positioned at a fixed distance of 45 cm from the participant’s eyes. Text was presented in a proportional serif font (MyeongjoChe). The display followed the MNREAD chart design guidelines, maintaining approximately 1.2× line spacing, standard inter-character spacing, and font scaling based on *x*-height to preserve equivalent visual angles across sizes. These parameters were consistent with the Korean MNREAD chart specifications [14]. The stimulus text consisted of 148 Korean syllables presented as unrelated words. This design was chosen deliberately to eliminate contextual cues, thereby ensuring that reading speed reflected oculomotor control and decoding rather than comprehension. The text was presented in 20-point font, black letters on a white background.

Participants were instructed to read the passage aloud at their natural pace. Each subject completed the reading task on two separate occasions, at least one day apart. This was done to minimize intra-individual variability and reduce potential learning or fatigue effects. The mean value of the two sessions was used for analysis, providing a more stable estimate of reading performance. The reading task began when the examiner pressed a button to start the reading screen and ended when the same button was pressed after finishing the passage. Reading speed was calculated in letters per second (LPS) by dividing the number of characters by the total time to completion.

During reading, binocular gaze position was continuously recorded at 40 Hz. Eye-tracking data were analyzed to compute fixation disparity indices. The eye-tracking procedure and the computation of the fixation disparity index were the same as those described in our previous validation study [15]. A nine-point calibration was performed before each session and repeated as needed until successful alignment was achieved. For the reading task, the fixation disparity index was defined as the proportion of recorded frames in which the interocular horizontal gaze difference exceeded the upper limit of the 95% confidence interval (CI), which was calculated from the orthophoric control group (n = 25) under identical conditions. The CI was computed for the entire control population rather than for each subject individually. This control-based CI was used as the baseline because perfect orthophoria is rare, and minor instrumental errors, such as corneal reflection loss or pupil center detection error, can occur. Thus, the CI from normal controls provided a realistic reference for defining normal fusion stability [15]. When the gaze-recognition rate of a trial fell below 50%, the calibration procedure was repeated before resuming the test. The mean usable frame rate was 83.0% in the intermittent exotropia group and 86.3% in the control group, showing no significant difference between groups (*p* = 0.397, Mann–Whitney U test). Each participant completed two reading sessions, and the mean of the two runs was used for subsequent analyses. Test–retest reliability, as assessed by the intraclass correlation coefficient (Kendall coefficient of concordance), was 0.747 for the fixation disparity index and 0.790 for letters per second (both *p* < 0.001).

### 2.4. Fusion Control Assessment

Conventional clinical scales were also applied for comparison with eye-tracking metrics. The Newcastle control score (NCS) was administered, which incorporates both parental observations and clinical assessment of manifest deviation under dissociative conditions [16,17]. In addition, the Mayo Clinic office-based scale was recorded, grading control on a 0–10 scale during repeated cover testing [18,19,20]. All assessments were performed by a trained single examiner (D.H.K) to reduce inter-observer variability.

### 2.5. Data Analysis

Data analysis was performed using SPSS version 26.0 (IBM Corp., Armonk, NY, USA). Continuous variables were summarized as means ± standard deviations, and categorical variables were expressed as counts. Group comparisons for continuous outcomes were conducted using the Mann–Whitney U test. Fisher’s exact test was used for categorical comparisons. Spearman’s rank correlation coefficient was used to examine correlations between reading speed and clinical characteristics.

## 3. Results

### 3.1. Clinical Characteristics of the Participants

A total of 50 individuals completed the study protocol, including 25 individuals in the intermittent exotropia group and 25 in the control group. Table 1 presents the demographic and baseline clinical characteristics of these participants.

The mean age of the intermittent exotropia group was 24.3 ± 6.6 years, ranging from 15.5 to 40.7 years. Thirteen were male and 12 were female. In contrast, the control group had a mean age of 26.8 ± 2.5 years, ranging from 21.4 to 30.9 years. This group consisted of 10 males and 15 females. The slightly higher proportion of females in the control group did not reach statistical significance (*p* = 0.157, Fisher’s exact test).

Distance BCVA in the intermittent exotropia group was 0.00 ± 0.01 LogMAR (range 0.00–0.02), and near BCVA was 0.00 ± 0.01 LogMAR (range 0.00–0.05). In the control group, distance BCVA was 0.00 ± 0.01 LogMAR (range: 0.00–0.05) and near BCVA was 0.00 ± 0.00 LogMAR.

The refractive error, expressed as the spherical equivalent, was −3.19 ± 2.74 diopters (range −9.88 to +1.81 D) in the intermittent exotropia group and −3.00 ± 2.89 diopters (range −8.94 to +0.69 D) in the control group.

Stereopsis, as measured by the Randot stereotest, averaged 1.95 ± 0.29 logArcsec (range 1.51–2.60) in the intermittent exotropia group and 1.58 ± 0.30 logArcsec (range 1.10–2.00) in the control group.

The average NPC was 8.7 ± 3.0 cm (range 3.5–26.0 cm) in the intermittent exotropia group and 7.9 ± 2.0 cm (range 5.0–13.0 cm) in the control group. The NPC difference between the intermittent exotropia and control groups was not significant. (*p* = 0.564) The NPA averaged 9.8 ± 3.7 cm (range 4.8–19.0 cm) in the intermittent exotropia group and 9.1 ± 2.0 cm (range 5.5–14.5 cm) in the control group.

Distance exodeviation was 21.1 ± 13.0 PD in the intermittent exotropia group, ranging from a minimum of 4 PD to a maximum of 50 PD. The control group recorded a mean of 0.2 ± 0.9 PD. Near exodeviation averaged 27.1 ± 10.0 PD (range 14–50 PD) in the intermittent exotropia group and 0.8 ± 1.4 PD (range 0–4 PD) in the control group. Both near and distance deviations were significantly different between groups (*p* < 0.001), as expected.

### 3.2. Reading Speed

**Figure 1:**
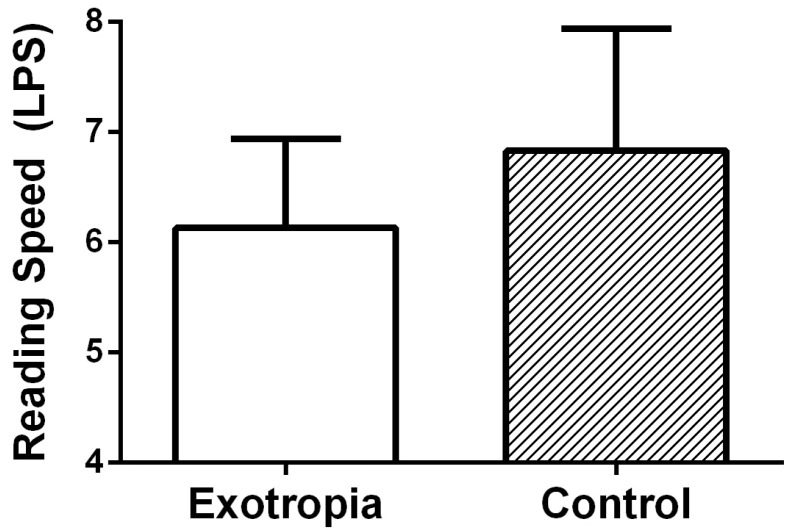
The reading speed (LPS: letters per second) was significantly slower in intermittent exotropia group (6.1 ± 0.8) than in the control group (6.8 ± 1.1, *p* = 0.015, Mann–Whitney U test).

Reading speed measured in letters per second (LPS) was slower in the intermittent exotropia group (*p* = 0.015, Cohen’s d = 0.7). The mean LPS in the intermittent exotropia group was 6.1 ± 0.8 (range 4.6–8.4) and the median was 6.1 (IQR 5.6–6.5), whereas the control group achieved 6.8 ± 1.1 (range 4.7–9.0) and the median was 6.6 (IQR 6.1–7.4). The distribution of LPS values in both groups is illustrated in Figure 1.

Reading performance and fixation disparity were further analyzed according to sex (Table 2). Within the intermittent exotropia group, reading speed did not differ significantly between males and females (6.1 ± 1.0 vs. 6.2 ± 0.6 letters per second, *p* = 0.856). In the control group, males tended to read slightly faster than females (7.3 ± 1.0 vs. 6.5 ± 1.1 letters per second), but the difference was not statistically significant (*p* = 0.104). Similarly, the fixation disparity score showed no significant sex difference within the intermittent exotropia group (36.4 ± 29.9 vs. 22.4 ± 27.4, *p* = 0.257).

### 3.3. Relationship Between Reading Speed and Related Factors

**Figure 2:**
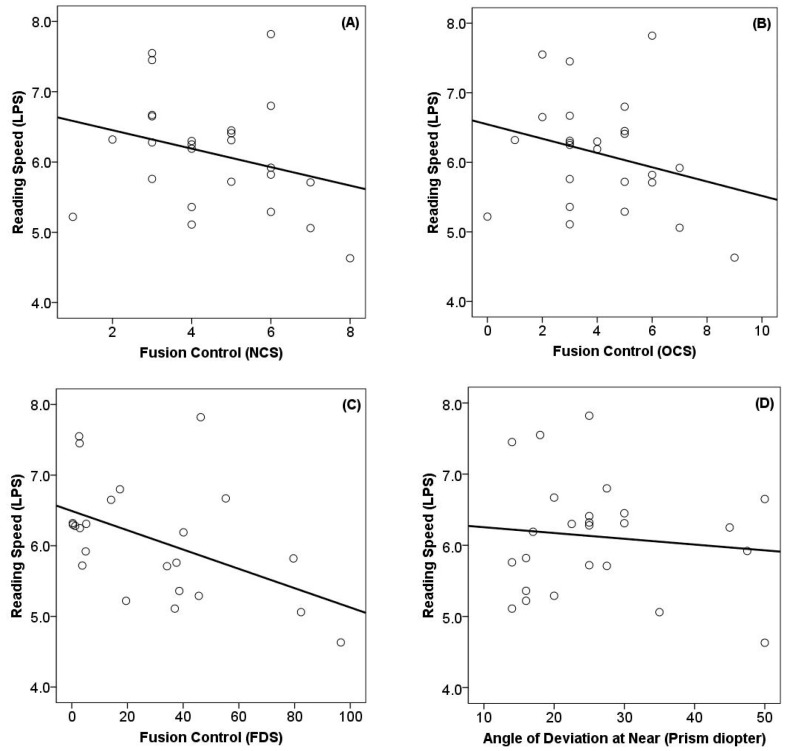
Relationship between reading speed and fusion control. The solid lines indicate the trend curve line. (**A**) Newcastle control score: r_s_ = −0.309, *p* = 0.132. (**B**) Mayo Clinic office-based scale: r_s_ = −0.267, *p* = 0.197. (**C**) Fixation disparity score: r_s_ = −0.458, *p* = 0.028. (**D**) Angle of near deviation: r_s_ = 0.051, *p* = 0.807). Abbreviation: LPS, letter per second; NCS, Newcastle control score; OCS, Mayo clinic office-based scale; FDS, fixation disparity score; r_s_, Spearman’s rank correlation coefficient.

The correlation analysis is shown in Figure 2. Reading speed did not significantly correlate with the Newcastle control score (rs = −0.309, *p* = 0.132) or the Mayo Clinic office-based scale (rs = −0.267, *p* = 0.197). Similarly, no meaningful relationship was observed between reading speed and the angle of near deviation (rs = 0.051, *p* = 0.807) or stereopsis (rs = −0.089, *p* = 0.694). By contrast, fixation disparity index obtained from the eye tracker during the reading task showed a negative correlation with reading speed (rs = −0.458, *p* = 0.028).

A generalized linear model including age, stereopsis, and refractive error as covariates was used to assess their effects on reading speed. None of these covariates showed a significant association with letters-per-second reading speed (age: B = −0.04, SE = 0.02, 95% CI: –0.08 to 0.00, *p* = 0.070; stereopsis: B = −0.05, SE = 0.06, 95% CI: −0.17 to 0.06, *p* = 0.361; refractive error: B = −0.37, SE = 0.45, 95% CI: −1.25 to 0.51, *p* = 0.409). These results indicate that age, stereopsis, and refractive error were not significant predictors of reading speed, suggesting that the group difference observed between intermittent exotropia and controls was independent of these covariates (Table 3).

## 4. Discussion

This study evaluated the reading performance of individuals with intermittent exotropia compared to controls, revealing a slower reading speed in the intermittent exotropia group during reading tasks. Correlations between reading speed and fusional control measures were explored, indicating a significant negative correlation with fixation disparity score.

Regarding the effects of intermittent exotropia on visual function, binocular inhibition was noted in 9.5% of 63 patients with intermittent exotropia during binocular vision, and a significant correlation exists between binocular visual acuity and better monocular visual acuity [21]. Measurements of reading speed could provide more information about visual function than visual acuity [22]. There have been several previous studies investigating various aspects of reading speed and its relationship to intermittent exotropia. Fang et al. [9] focused on school-aged children with intermittent exotropia and found that age, sex, and stereo function were associated with differences in reading speed. Yang et al. [10] highlighted broader deficits in visual-motor integration, vocabulary, and attention that predicted reading performance. Ridha et al. [23] showed that strabismus surgery in children resulted in improved reading ability, suggesting its potential impact on academic performance. Kim and Choi [24] found that children between 7 and 12 years of age with intermittent exotropia had significantly slower reading speeds, highlighting the need for early intervention and monitoring. Rakshit et al. [25] also found poorer fine motor skills in addition to slower reading performance in patients with strabismus. More recently, Wei et al. [26] demonstrated that children with intermittent exotropia exhibited impairments in both visual and auditory attention, which may partly explain difficulties with sustained reading tasks. Lamoureux et al. [27] further confirmed that binocular reading speed is reduced across strabismus populations. Taken together, these findings and our study strengthen the conclusion that reduced reading ability in intermittent exotropia is not an isolated observation limited to a single study but rather a consistent pattern observed across different age groups, cultural contexts, and methodological approaches.

There are only two studies that evaluated binocular coordination using video-oculography [11,12]. Hirota et al. [11] showed that saccadic disconjugacy was associated with re-reading the same line in patients with convergence insufficiency type intermittent exotropia. Later, they found that the reading speed was negatively correlated with the proportion of monocular viewing in the intermittent exotropia group during smartphone reading of vertical scripts [12]. Interestingly, no participants recognized blurred vision or diplopia during smartphone reading. Our study also objectively showed correlations between reading speed and fusion control measures, indicating a significant negative correlation with fixation disparity score. It demonstrates that even without conscious awareness of blur or diplopia, patients may adopt a monocular strategy that slows down reading efficiency [12]. Our present study, which used infrared eye tracking, supports these findings by confirming that fixation disparity indices are objectively linked to reduced reading speed.

Age is a common factor that affects reading speed [24]. Calabrèse et al. [28] reported that reading speed increases until 16 years of age, reaches an equilibrium at age 16, and then declines by 40 years of age. Chen et al. [29] reported that reading speed increases until 19 years of age, reaches an equilibrium at age 20, and then decreases by 40 years of age. In other words, reading speed reaches an equilibrium after teenage years and then declines after middle age. In our study, two age-matched groups were compared between 15 years of age and 40 years of age, which is when reading speed is known to equilibrate, and found that objective fusion control scores affected reading speed in intermittent exotropia.

There is some debate as to whether ocular misalignment itself or the angle of deviation affects reading speed. Dysli et al. [30] measured reading performance after inducing horizontal and vertical phoria by applying prisms to 16 healthy subjects and reported that phoria did not affect reading performance. Latvala et al. [31] compared children with dyslexia and normal children and found no significant difference in the strabismus angle between the two groups but reported more children in the dyslexia group with NPC of 8 cm or greater. However, in our study, the lack of correlation between deviation angle and reading speed (*p* = 0.807) supports the idea that alignment magnitude alone does not predict functional impairment, and that dynamic binocular coordination plays a more decisive role.

Previous studies have suggested possible sex-related differences in reading speed and eye-movement behavior, with females generally showing longer fixation durations or slower overall reading rates, possibly reflecting greater attention to linguistic context and comprehension [32]. In our study, however, no significant sex difference in reading speed was observed in either group. One possible explanation is that the reading material consisted of context-free, short sentences rather than continuous text, reducing the influence of higher-level linguistic processing. Future studies using more naturalistic reading passages may better reveal whether subtle sex-related differences in reading strategy or binocular coordination exist.

Clinically, reduced reading speed may contribute to academic difficulties in intermittent exotropia and highlight the importance of early monitoring and potential intervention. Prior work has shown that strabismus surgery can improve reading ability in children [23], and Perrin Fievez et al. [33] suggest post-surgical improvements in eye movements during reading. Feuillade et al. [34] further noted that surgery may enhance learning abilities, reinforcing the potential educational benefits of timely intervention. Counseling for families should therefore include discussion of functional limitations in reading, in addition to psychosocial concerns. Furthermore, incorporating eye tracker–based assessments could refine the evaluation of fusion control and help identify patients most at risk for functional impairment.

Beyond the clinical measures, the present results carry potential educational implications. Reading fluency is a fundamental prerequisite for academic achievement, and slower reading in intermittent exotropia patients may contribute to increased fatigue during school tasks. A large-scale population-based analysis has also shown that adolescents with strabismus may experience not only visual problems but also poorer academic outcomes and psychosocial disadvantages [27]. Our data therefore support the need to incorporate functional reading assessments into both clinical follow-up and educational support strategies.

Another important area relates to digital reading environments. Unlike traditional print materials, reading on smartphones or tablets often requires shorter viewing distances and smaller font sizes, which can aggravate binocular instability. A recent eye-tracking study reported measurable digital reading fatigue in strabismus [35]. Moreover, the emergence of artificial intelligence and wearable eye-tracking platforms now makes it possible to detect intermittent strabismus with high accuracy in naturalistic conditions [36]. These developments suggest that future research should combine digital reading tasks with automated assessment to better capture the everyday challenges faced by patients with intermittent exotropia.

There are some limitations in this study. Firstly, patients with amblyopia with a difference in visual acuity of more than two lines were excluded. Some children with amblyopia showed below-average oral reading ability despite good treatment outcomes [37]. Therefore, the results of this study cannot be extrapolated to those patients with amblyopia. Secondly, this study primarily focused on reading speed and did not address reading comprehension or learning ability. Future work should include standardized comprehension assessments to determine whether slower reading in intermittent exotropia directly leads to reduced understanding of the material. Thirdly, our findings may be difficult to apply to smartphone reading, which is commonly used nowadays, because the viewing distance of a smartphone is closer than that of a hardcopy text [38,39]. Lastly, reading silently is faster than reading aloud for skilled readers. We instructed the participants to read aloud to verify their reading, which may introduce differences compared to reading silently.

## 5. Conclusions

In summary, patients with intermittent exotropia exhibited significantly slower reading speeds compared with age-matched controls, and fixation disparity measured with an eye tracker emerged as a clinical factor that significantly correlated with reading performance. These results reinforce the concept that strabismus influences functional domains of daily life, including reading fluency.

In conclusion, the present study provides new, objective evidence that intermittent exotropia measurably affects reading fluency. Future research should expand beyond reading speed to include reading comprehension, cognitive load, and fatigue assessments, especially in digital environments where most adolescents and young adults now spend significant time.

## Figures and Tables

**Table 1:** Baseline characteristics of all participants.

	Intermittent Exotropia Group (n = 25)			Control Group (n = 25)				
Characteristics	Mean ± SD (Min~Max)	Median	IQR	Mean ± SD (Min~Max)	Median	IQR	*p* Value	Cohen’s D
Sex (M:F)	13:12			10:15			0.157 *	
Age at examination (y)	24.3 ± 6.6 (15.5~40.7)	24.2	19.3~26.0	26.8 ± 2.5 (21.4~30.9)	26.5	24.9~29.0	0.095 ^†^	0.5
Distance BCVA (LogMAR)	0.00 ± 0.01 (0.00~0.02)	0.00	0.00~0.00	0.00 ± 0.01 (0.00~0.05)	0.00	0.00~0.00	0.428 ^†^	0.0
Near BCVA (LogMAR)	0.00 ± 0.01 (0.00~0.05)	0.00	0.00~0.00	0.00 ± 0.00 (0.00~0.00)	0.00	0.00~0.00	0.317 ^†^	0.0
Refractive errors, SEQ (D)	−3.19 ± 2.74 (−9.88~+1.81)	−2.50	−5.10~−1.34	−3.00 ± 2.89 (−8.94~+0.69)	−2.98	−4.69~−0.47	0.720 ^†^	0.1
Stereopsis (LogArcsec)	1.95 ± 0.29 (1.51~2.60)	2.00	1.70~2.00	1.58 ± 0.30 (1.10~2.00)	1.58	1.35~1.80	<0.001 ^†^	1.3
NPC (cm)	8.7 ± 3.0 (3.5~26.0)	7.0	6.0~10.8	7.9 ± 2.0 (5.0~13.0)	7.0	6.0~9.3	0.564 ^†^	0.3
NPA (cm)	9.8 ± 3.7 (4.8~19.0)	10.0	6.4~11.6	9.1 ± 2.0 (5.5~14.5)	9.5	7.3~10.5	0.655 ^†^	0.2
Distance deviation (PD)	21.1± 13.0 (4~50)	20.0	7.0~28.8	0.2 ± 0.9 (0~4)	0.0	0.0~0.0	<0.001 ^†^	2.3
Near deviation (PD)	27.1 ± 10.0 (14~50)	25.0	16.5~30.0	0.8 ± 1.4 (0~4)	0.0	0.0~0.0	<0.001 ^†^	3.7

M = male; F = female; n = numbers; y = years; BCVA = Best corrected visual acuity; LogMAR = logarithm of the minimum angle of resolution; SEQ = Spherical equivalent; D = diopters; NPA = Near point of convergence; NPC = Near point of accommodation; PD = prism diopters. * *p* value by Fisher’s exact test, ^†^ Mann–Whitney U test.

**Table 2:** Reading speed and fusion disparity score by sex.

	Male		Female		
	Mean ± SD (Min~Max)	Median (IQR)	Mean ± SD (Min~Max)	Median (IQR)	*p* Value
LPS_IXT group	6.1 ± 1.0 (4.6~8.4)	6.0 (5.5~6.6)	6.2 ± 0.6 (5.6~7.4)	6.2 (5.6~6.5)	0.856
LPS_Control group	7.3 ± 1.0 (5.5~9.0)	7.2 (6.8~7.9)	6.5 ± 1.1 (4.7~9.0)	6.5 (6.1~6.7)	0.104
FD score_IXT group	36.4 ± 29.9 (2.9~96.6)	34.2 (14.1~46.3)	22.4 ± 27.4 (0.4~82.3)	26.5 (14.4~39.5)	0.257

SD = standard deviation; Min = minimum; Max = maximum; IQR = interquartile range; LPS = letters per second; IXT = intermittent exotropia; FD = fixation disparity. *p* value by Fisher’s exact test.

**Table 3:** Generalized linear model for reading speed (letters per second).

Predictor	B (Estimate)	SE	95% CI	Wald χ^2^	*p* Value
Age (y)	−0.04	0.02	−0.08 to 0.00	3.29	0.070
Stereopsis (LogArcsec)	−0.05	0.06	−0.17 to 0.06	0.83	0.361
Refractive errors (D)	−0.37	0.45	−1.25 to 0.51	0.68	0.409

y = years; D = diopters; SE = standard error; CI = confidence interval.

## Data Availability

The raw data supporting the conclusions of this article will be made available by the authors on request. For data requests, please contact 98614@snubh.org.
